# Clinical and Pathological Characteristics of Chronic Otomastoiditis: A Retrospective Analysis of Risk Factors, Outcomes, and Antibiotic Resistance Patterns

**DOI:** 10.3390/healthcare12242518

**Published:** 2024-12-12

**Authors:** Cristina Popescu, Alin Iulian Silviu Popescu, Renata Maria Văruț, Mihaela Popescu, Carmen Elena Niculescu, Cristina Elena Singer

**Affiliations:** 1 ENT County Hospital Craiova, Discipline of Anatomy, Department of Anatomy, University of Medicine and Pharmacy, 200349 Craiova, Romania; cristina.popescu@umfcv.ro; 2 Department of Internal Medicine, University of Medicine and Pharmacy of Craiova, 200349 Craiova, Romania; 3 Department of Research Methodology, Faculty of Pharmacy, University of Medicine and Pharmacy of Craiova, 200349 Craiova, Romania; 4 Department of Endocrinology, University of Medicine and Pharmacy of Craiova, 200349 Craiova, Romania; mihaela.popescu.e@umfcv.ro (M.P.); drcarmen88@yahoo.com (C.E.N.); 5 Department of Mother and Baby, University of Medicine and Pharmacy of Craiova, 200349 Craiova, Romania; cristina.singer@umfcv.ro

**Keywords:** otomastoiditis, chronic otitis media, hearing loss, antibiotic resistance, clinical symptomatology, radiological examination, audiometry, cholesteatoma, retrospective study, rural vs. urban healthcare

## Abstract

**Background/Objectives:** Chronic otomastoiditis is a complex inflammatory condition frequently associated with delayed diagnosis, inadequate antibiotic use, and healthcare disparities. This study aimed to analyze the clinical, demographic, and microbiological characteristics of chronic otomastoiditis and its complications over a 10-year period in rural versus urban populations. **Methods:** This retrospective study included 292 patients with chronic otomastoiditis admitted to the ENT Clinic of Craiova County Emergency Clinical Hospital from 2013 to 2023. Data were collected on patient demographics, clinical presentations, imaging findings, audiometry, bacteriological profiles, and surgical outcomes. Statistical analyses were conducted to identify risk factors and patterns of antibiotic resistance. **Results:** Urban patients represented 60.27% of cases, while rural patients (39.73%) presented later with more advanced disease. Cholesteatoma was identified in 49.31% of cases, frequently associated with hearing loss and structural complications. Significant antibiotic resistance was noted for *Streptococcus pneumoniae* and *Staphylococcus aureus*, with high resistance rates to amoxicillin and amoxicillin–clavulanate. Surgical interventions, primarily mastoidectomy, were associated with varied recovery rates and complications. **Conclusions:** Chronic otomastoiditis is influenced by healthcare accessibility and antibiotic resistance. Early diagnosis, antibiotic stewardship, and targeted surgical interventions are critical in managing this condition, particularly in underserved rural populations. Public health efforts should focus on improving healthcare accessibility to mitigate long-term complications.

## 1. Introduction

Otomastoiditis is a complex inflammatory condition involving the middle ear and mastoid air cells, often resulting from inadequately treated chronic otitis media. Globally, the disease has a significant impact on both healthcare costs and patients’ quality of life due to its potential complications and prolonged course. Otomastoiditis can occur in both acute and chronic forms, with the chronic form being more prevalent in clinical practice [1]. Globally, otomastoiditis imposes a significant clinical and economic burden, particularly in underdeveloped regions where its chronic form frequently leads to prolonged disease progression and complications such as hearing loss [2]. Otomastoiditis is estimated to contribute to 5–10% of hearing loss cases worldwide. Emerging pathogens, including resistant bacterial strains and viral agents, have been increasingly implicated in the chronicity of ear diseases such as otomastoiditis, complicating treatment and leading to more persistent infections [3].

Antibiotic resistance is increasingly recognized as a critical issue in the management of otomastoiditis, particularly in chronic cases. The widespread misuse and overprescription of antibiotics have led to the emergence of resistant strains of bacteria, such as *Streptococcus pneumoniae*, *Staphylococcus aureus*, and *Pseudomonas aeruginosa*, which are commonly implicated in both acute and chronic otomastoiditis [4]. Studies have demonstrated a high rate of resistance to commonly used antibiotics, including amoxicillin and amoxicillin–clavulanate, making standard empirical therapies less effective. This resistance complicates treatment, often necessitating the use of more potent antibiotics like fluoroquinolones or third-generation cephalosporins, which come with their own risks of further resistance development and adverse effects [5].

Bacteriological analysis plays a key role in identifying the specific pathogens responsible for otomastoiditis and determining their antibiotic sensitivity. Routine cultures of ear discharge help guide targeted therapy, allowing clinicians to select antibiotics that are more likely to be effective, thereby reducing the likelihood of treatment failure. However, the growing prevalence of multi-drug-resistant organisms in chronic cases underscores the need for careful antibiotic stewardship and early intervention to prevent the progression of disease [6].

Cholesteatoma is a well-known complication of chronic otomastoiditis, characterized by the abnormal accumulation of keratinizing epithelium in the middle ear and mastoid, which leads to the destruction of surrounding bone and tissue. This process not only causes conductive hearing loss but also increases the risk of serious complications such as intracranial infections and facial nerve paralysis if left untreated [7]. Cholesteatoma often requires surgical management, including mastoidectomy, to remove the lesion and prevent further damage. Despite advances in surgical techniques, the presence of cholesteatoma significantly complicates treatment outcomes, particularly in patients with concurrent antibiotic-resistant infections [8].

Effective management of otomastoiditis, especially in the presence of cholesteatoma and antibiotic-resistant bacteria, necessitates a multidisciplinary approach that integrates both medical and surgical interventions. Early identification of resistant pathogens through bacteriological studies, coupled with appropriate surgical management, remains crucial to improving patient outcomes and preventing long-term complications [9]. As antibiotic resistance continues to rise globally, there is an urgent need to refine treatment protocols and promote the judicious use of antibiotics in both outpatient and hospital settings to curb the growing public health threat posed by resistant infections [10].

It is essential to understand the epidemiology of otomastoiditis to grasp the broader clinical significance of the disease. While acute episodes of otomastoiditis are less frequent, chronic infections pose a significant burden on patients and healthcare systems [11]. Chronic otomastoiditis, especially, is known for its persistence, leading to complications such as hearing loss and even life-threatening conditions if untreated. Historically, before the introduction of antibiotics, otomastoiditis was a frequent complication of acute otitis media, affecting up to 50% of patients with untreated ear infections. However, in the era of modern antibiotic treatments, its incidence has declined dramatically [12]. Despite this, the condition has seen a resurgence in recent years, particularly in chronic cases, due to factors such as improper use of antibiotics and increasing rates of antibiotic-resistant bacteria [13].

There is a notably high frequency of otomastoid involvement in populations with low economic status and inadequate healthcare systems, in contrast to the rarity or even near absence of the condition in populations with a high economic level. Otomastoiditis is described in both sexes and across all age groups. However, it occurs more frequently in children, particularly in very young ones. One study reports that 47% of acute otomastoiditis cases occur in infants [14,15,16]. Acute otomastoiditis has been described as occurring bilaterally and even in patients with aural atresia. Clinically, a diagnosis of mastoiditis is suspected whenever there is no improvement during the course of acute otitis media following treatment (myringotomy and antibiotics), or when there is a worsening of local and general symptoms after a temporary improvement [17,18]. Otomastoiditis is often accompanied by characteristic ear pain, sometimes radiating to surrounding regions, particularly when mastoid pus is retained. The pulsatile pain sensation is pathognomonic for the lack of drainage of the mastoid empyema. The persistence of pain and pulsatile sensations after tympanic paracentesis or spontaneous perforation is a decisive sign for diagnosing mastoiditis. Vertigo may also be present, indicating the possible spread of infection. Hearing loss is conductive in nature and highly variable [19,20]. On the other hand, the general signs are very important for diagnosis, often showing a patient who is fatigued, febrile or subfebrile, anorexic, pale, with an altered facial appearance. Less commonly, the onset may be marked by chills and high fever, symptoms more frequently observed in young children. The diagnosis is supported by objective signs obtained through inspection and palpation, otoscopy, and functional examination [21,22]. The progression to chronic mastoiditis is influenced by factors such as incomplete surgery, persistent otorrhea, patient conditions (e.g., diabetes, allergy), the anatomical structure of the mastoid, and the etiology and treatment of acute otitis [23,24]. Acute suppurative otomastoiditis typically involves mastoid abscesses, while chronic forms result in osteitic lesions, necrosis, or cholesteatoma in the antromastoid region. Chronic mastoiditis reflects a persistent bacterial infection of the mastoid. Recent diagnostic methods prioritize high-resolution CT scans, and treatment includes advanced antibiotics and surgical interventions like mastoidectomy [25,26,27].

This study addresses gaps in understanding the demographics, antibiotic resistance patterns, and the long-term outcomes of otomastoiditis patients, emphasizing distinctions between rural and urban populations.

## 2. Materials and Methods

### 2.1. Study Design and Clinical Evaluation Protocol

This study is a retrospective clinico-statistical analysis conducted over a 10-year period (2013–2023). It involved reviewing the medical records of 292 patients diagnosed with chronic otic pathology admitted to the ENT Clinic of the County Emergency Clinical Hospital in Craiova. The patients were selected retrospectively from the total pool of admissions during this period, based on their confirmed diagnosis of ENT conditions. Patients aged 10–70 years with a confirmed otomastoiditis diagnosis and documented treatment history, including antibiotic prescriptions and previous ear surgeries, were included. The exclusion criteria involved patients with immune-compromising conditions to ensure consistency in disease progression analysis.

The diagnosis of each patient was established through the evaluation of existing data in the medical records, which included anamnesis, physical and functional ENT examinations, and relevant paraclinical investigations. Data were extracted from multiple sources such as the hospital admission registers, surgical intervention logs, pathology and laboratory reports, and imaging examinations, including mastoid X-rays and CT/MRI scans. The selection of diagnostic and paraclinical tests was based on their relevance to the comprehensive evaluation of chronic otomastoiditis. Bacteriological analysis was chosen to identify the pathogens most commonly implicated in the disease and to determine their antibiotic resistance profiles, enabling targeted treatment. High-resolution CT scans and MRI were used to detect critical structural damage, including cholesteatoma, ossicular erosion, and labyrinthine involvement, aiding surgical planning. Tonal audiometry was performed universally to assess the hearing loss severity and type, aiding in the evaluation of the disease’s impact on auditory function. Histopathological examinations of intraoperatively collected tissue fragments were conducted to confirm the presence of cholesteatoma or other pathological changes, ensuring diagnostic accuracy. This comprehensive review was aimed at accurately capturing the clinical profile and outcomes of the patients over the study period.

The retrospective nature of the study meant that no new clinical data were collected; rather, the existing records were thoroughly analyzed. Special emphasis was placed on the identification of primary symptoms, including the onset and progression of otic pathology, along with the patient’s history of ear-related conditions. For each patient, a standardized set of key diagnostic signs was reviewed, including the date and duration of ear discharge, the appearance and quantity of otic secretion, external auditory canal characteristics, the tympanic membrane status (e.g., congestion, infiltration, or pulsatile pus), the retroauricular tissue condition and auditory function status, and general health indicators (fever, fatigue, insomnia, pallor). Additionally, any systemic conditions affecting the auditory-vestibular system were documented. The ENT clinical examination records followed established medical standards and involved detailed buccopharyngoscopy, anterior and posterior rhinoscopy, and otoscopy. The collection of auricular secretions for the purpose of identifying pathogens was performed by swabbing with a cotton-tipped stylus, with the storage and transport of the collected secretions conducted under strict sterile conditions, in accordance with established procedures.

The clinical data were analyzed retrospectively, with no active involvement of patients during the study period. Ethical approval was obtained for the retrospective analysis of patient records.

### 2.2. Statistical Data Analysis

All statistical analyses were conducted retrospectively on the patient data extracted from medical records. The data were compiled into tables and analyzed using Microsoft Excel (2007). Graphical representations of variability were created to illustrate the findings.

For the statistical interpretation, Chi-square and Cramer’s V tests were employed to assess the dependence between different classification factors. The Chi-square test was used to evaluate the relationships between categorical variables, such as patient demographics and clinical outcomes, and the level of significance was determined at three thresholds:

- *p* < 0.05: Statistically significant (95% confidence);
- *p* < 0.01: Statistically significant (99% confidence);
- *p* < 0.001: Highly significant (99.9% confidence).

The results were interpreted with respect to whether the associations between variables, such as patient age or treatment outcomes, were significant. Cramer’s V test was used to determine the strength of associations in larger contingency tables, providing a more detailed understanding of the relationships between nominal factors. To enhance the robustness of the statistical analysis and identify the predictors of outcomes, a multivariate analysis was performed using logistic regression models. These models were employed to assess the association between patient demographics (age, gender, rural vs. urban origin) and clinical factors (duration of disease, antibiotic resistance, presence of complications) with key outcomes such as recurrence rates and treatment success. The results were expressed as odds ratios (ORs) with 95% confidence intervals (CIs), and a significance threshold of *p* < 0.05 was applied. This approach provided a deeper understanding of the independent contributions of each variable to the observed clinical outcomes, complementing the univariate analyses. 

## 3. Results

Upon analyzing the patients admitted to the ENT Clinic of the County Emergency Clinical Hospital in Craiova during the years 2013–2023, we observed a total of 22,750 admissions for various conditions. Among these, 292 patients (1.28%) were diagnosed with chronic otomastoiditis.

Among the total cohort of 292 patients, a slightly higher proportion were male, accounting for 164 cases (56.16%), compared to females, who constituted 128 cases (43.84%). This gender disparity may be attributed to differences in exposure to risk factors, such as occupational hazards or environmental conditions, which could disproportionately affect male patient. Considering the age and sex of the patients in the study cohort, we observed a higher percentage of male patients in most age groups compared to females, with the exception of the 41–50 age group, where female patients predominated. The age groups most affected by the suppurative otomastoid process, namely young adults aged 21–30 and adults aged 51–60, had the highest number of male patients. Using the Chi-square test, we obtained a value of 2.41, which is lower than the threshold value of 14.07 at the 95% confidence level for 8 × 2 tables. The corresponding *p*-value for this result is 0.94, significantly higher than 0.05, indicating that we could not statistically demonstrate any association between the age and sex of patients with otomastoiditis. The results of this study indicated a higher proportion of patients from urban areas (60.27%) compared to those from rural areas (39.73%).

Among adults, we observed a downward trend in the number of cases, with higher values around 19.86% in young adults and 14.38% in the 41–50 years age group. A significant increase of approximately 7% was noted in the 51–60 years age group. We observed a small difference of about 8% between the second age group (11–20 years) and the third age group (21–30 years), with a notable increase in the number of cases in the third age group (Table 1).

Among the total number of patients with otomastoiditis, there were 66 students (22.60%), 100 patients with secondary or elementary education (34.24%), 30 patients with higher education (10.27%), 20 unemployed patients (6.84%), and 76 retirees (26.02%).

A detailed anamnesis of the patients revealed that the duration of ear discharge ranged between 0 and 3 months in 16 patients (5.47%), 3 months to 1 year in 10 patients (3.42%), 1 to 10 years in 22 patients (7.53%), 10 to 20 years in 102 patients (34.93%), and over 20 years in 142 patients (48.63%) (Table 2).

Both forms of otomastoiditis, those with a progression of less than 3 months and those with a duration of more than 3 months, predominantly affected male patients, with the overwhelming majority of cases belonging to the chronic form of the disease. The small percentage of patients presenting with forms that evolved in less than 3 months did not allow for a generalization of the results obtained within this category. From the medical history of patients with chronic otomastoiditis, it was noted that many had a longstanding history of ear issues, with numerous episodes of exacerbation and a poor response to antibiotic treatment administered on an outpatient basis. In cases of otomastoiditis with a duration of less than 3 months, patients from rural areas predominated, compared to cases with a longer disease duration, exceeding 3 months, where the number of patients from urban areas was higher. However, the small number of patients in the category of otomastoiditis with a progression of less than 3 months did not allow for the generalization of the results obtained within this group (Table 2).

The personal medical history related to otic pathology revealed that the vast majority of patients (94.52%) experienced numerous episodes of recurrent otitis. Recurrences occurred at various intervals, accompanied by exacerbations of symptoms and hearing loss acknowledged by the patients. A thorough collection of data indicated not only the clinical, subjective, and objective progression of the disease but also a poor response to outpatient medical treatments.

Anamnestic data showed that a small percentage of patients (5.47%) had no history of suppurative otic conditions, with the onset of the disease described as occurring a few weeks or months before, coinciding with the development of complications.

Only 6.84% of the patients had a history of surgery, specifically partial petro-mastoidectomy, but the subsequent course was unfavorable in these cases, eventually requiring radical surgical therapy, which led to a good outcome.

The initial symptoms of otomastoiditis were the classic ones: otorrhea, otalgia, hearing loss, fever, and headache, while in chronic cases, the symptoms included persistent fetid otorrhea and hearing loss.

Thus, the subjective clinical symptomatology included: fetid otorrhea in 268 patients (91.78%), otalgia in 170 patients (58.21%), hearing loss in 292 patients (100%), general malaise in 86 patients (29.45%), fever/chills in 36 patients (12.32%), nausea, vomiting in 4 patients (1.36%), and vertigo in 34 patients (11.64%).

The occurrence of chronic forms and complications was observed in the patients with associated chronic general conditions, including hypertension in 114 patients (39.04%), diabetes mellitus in 64 patients (21.91%), liver diseases in 46 patients (15.75%), and gastrointestinal disorders in 36 patients (12.32%).

The otoscopic examination of the patients in the study revealed purulent secretions in the external auditory canal in 268 patients (91.78%), polypoid formations in 84 patients (28.76%), cholesteatoma lamellae in 144 patients (49.31%), external auditory canal collapse in 14 patients (4.79%), Gellé fistula in 10 patients (3.42%), epitympanic perforation in 144 patients (50.34%), mesoepitympanic perforation in 94 patients (32.87%), and mesotympanic perforation in 48 patients (16.78%).

The anatomoclinical forms of otomastoiditis encountered in this study were as follows: simple cholesteatomatous forms in 10 patients (3.42%), suppurative cholesteatomatous forms in 110 cases (37.67%), simple polypoid forms in 16 cases (5.47%), suppurative polypoid forms in 52 cases (17.80%), simple suppurative forms in 66 cases (22.60%), and combined suppurative polypoid cholesteatomatous forms in 38 cases (13.01%) (Table 3).

In the studied cohort, we observed 258 patients (96.27%) with unilateral ear suppuration and 10 patients (3.73%) with bilateral ear suppuration. Out of the total number of patients with otomastoiditis, 152 were diagnosed with left-sided otomastoiditis, 130 with right-sided otomastoiditis, and 10 patients were diagnosed with bilateral otomastoiditis.

The anatomoclinical form of otomastoiditis was determined through a histopathological examination of intraoperatively collected tissue fragments. Therefore, the postoperative hospitalization period did not correlate with the anatomoclinical form; the hospitalization period after total petro-mastoidectomy was approximately two weeks for the patients without major complications (Table 4).

Postoperatively, meticulous daily care of the petro-mastoidectomy cavity was performed, involving the application of a zinc oxide, chloramphenicol, and hydrocortisone acetate ointment. The petro-mastoidectomy cavity in the operated patients showed favorable healing, with epithelialization occurring within two weeks. The patients with intracerebral complications required a longer hospitalization period, depending on the severity of the intracerebral lesions.

Performing the Chi-square test, we obtained a value of 42.520, which, for 4 × 2 tables, is significantly higher than the minimum confidence thresholds of 95% (7.82), 99% (11.35), and 99.9% (16.27). The corresponding *p*-value for this result is 0.00001, indicating a very high level of statistical significance.

The result of the Cramer’s V test, 0.453, indicates a strong association between the categories of the two factors, the anatomoclinical form, and the duration of hospitalization.

The complementary paraclinical investigations used in establishing the diagnosis of otomastoiditis included classic radiological examination and tonal audiometry, both of which were performed in all 292 cases (100%). Additionally, bacteriological examination with antibiogram of ear secretions was conducted in 268 cases (91.78%), and a histopathological examination of the polyps, cholesteatomas, or mucosal fragments collected intraoperatively was performed in 292 patients (100%). Imaging examinations (CT/MRI) were conducted in the 148 patients (50.68%) for whom complications were suspected.

Table 5 provided a comparative analysis of imaging characteristics, complications, and prevalent age groups across different types of otomastoiditis. Cholesteatomatous otomastoiditis was marked by significant bone erosion and the presence of cholesteatoma, with labyrinth involvement frequently observed as a complication. This type predominantly affected individuals in the 30–50 age range, indicating a possible correlation between age-related structural vulnerabilities and cholesteatoma formation. Polypoid otomastoiditis primarily presented with soft tissue swelling on imaging and was notably free from severe complications. This type appeared more commonly in younger adults aged 20–40, suggesting a milder progression in this age demographic. Suppurative otomastoiditis was characterized by mastoid air cell opacification, which often led to serious complications such as intracranial spread. This form predominantly affected individuals between 40 and 60 years of age, aligning with the tendency for more advanced disease progression and potential intracranial involvement in older age groups.

In our study, postoperative recurrence rates were carefully analyzed to identify timing and contributing factors. Mastoidectomy was the most common intervention, accounting for 68% of the cases. It had a complication rate of 21.7% and a recurrence rate of 18.5% within 24 months, with a recovery rate of 76.3%. Tympanoplasty combined with mastoidectomy was performed in 32% of the cases and demonstrated a lower complication rate of 14.9% and a recurrence rate of 10.6%. This approach yielded a higher recovery rate of 83.2%, suggesting its effectiveness in achieving long-term patient recovery. Simple tympanoplasty, accounting for 8% of cases, had the lowest complication rate (8.3%) and recurrence rate (4.2%), with a high recovery rate of 91.7%, indicating its success in less complex cases with a lower recurrence risk. Revision surgery was required in 5% of the cases with recurrent disease, showing the highest complication rate of 33.3% and a recurrence rate of 26.7%, with a recovery rate of 73.3%. These outcomes reflect the increased risk associated with revision surgeries for recurrent otomastoiditis (Table 6). A long-term follow-up revealed that recurrences typically occurred within 12 to 24 months post-surgery, often presenting with persistent otorrhea, hearing loss, or evidence of unresolved infection. The patients with co-morbid conditions such as diabetes or chronic respiratory diseases were at a higher risk for recurrence, particularly in cases involving antibiotic-resistant pathogens. These findings underscore the importance of targeted post-surgical monitoring to minimize recurrence and optimize outcomes.

Mild hearing loss (20–40 dB) was observed in 25% of the patients, with a mean age of 35 years. Cholesteatoma was present in 10% of these cases, indicating a limited association with this level of hearing impairment. Moderate hearing loss (40–60 dB) affected 40% of the patients, with a mean age of 45 years. Cholesteatoma was present in 30% of the cases, suggesting a higher likelihood of disease progression associated with moderate hearing loss. Severe hearing loss (60–80 dB) was noted in 20% of the patients, with an average age of 50 years. Cholesteatoma presence increased to 50%, reflecting a strong association between severe hearing impairment and cholesteatoma development.

Profound hearing loss (>80 dB) impacted 15% of the patients, who had a mean age of 60 years. In this group, cholesteatoma was found in 70% of the cases, indicating that profound hearing loss is highly correlated with advanced disease and structural complications.

In a total of 268 patients, ear secretion samples were collected to determine the microbial pathology involved in the suppurative process and the antibiotic sensitivity for the appropriate application of medical treatment. Based on the microbiological examination data, the following pathogens were identified in this study: *Streptococcus pneumoniae* in 36.56% of the cases, *Staphylococcus aureus* in 13.43%, and *Pseudomonas aeruginosa* in 11.19%. A smaller proportion of cases recorded *Proteus mirabilis* in 5.22% of the cases and *Haemophilus influenzae* in 6.71% of the cases. In the cases where bacteriological tests did not detect pathogenic flora (11.94%), the same type of antibiotic treatment that had been prescribed prior to the patient’s outpatient visit was continued (Table 7).

A detailed patient history revealed the prolonged use of various antibiotics to treat repeated episodes of otitis media or other acute upper respiratory tract infections, which could explain the recent increase in antibiotic resistance. As demonstrated in this study, as well as in other specialized works, routine antibacterial therapy for otitis is not an absolute guarantee against the development of mastoiditis and its complications. In some patients, routine antibiotic treatment may delay or mask mastoiditis and its associated complications. Amoxicillin shows resistance in 32 patients, particularly against *Streptococcus pneumoniae* and *Staphylococcus aureus*, suggesting that this common antibiotic may no longer be a reliable first-line treatment for these pathogens. Similarly, amoxicillin–clavulanate, which is often used due to its broad spectrum, was ineffective in 23 cases, with resistance primarily coming from *Pseudomonas aeruginosa* and *Staphylococcus aureus*. Ceftriaxone, another commonly used antibiotic, was found to be ineffective in 16 patients, especially against *Haemophilus influenzae* and *Proteus mirabilis*. This is particularly concerning as ceftriaxone is typically reserved for more severe infections. Ciprofloxacin, frequently used to combat a wide range of bacterial infections, showed resistance in 13 cases, with *Pseudomonas aeruginosa* and *Staphylococcus aureus* being the main resistant organisms. In addition, azithromycin faced resistance in 11 cases, predominantly from *Staphylococcus aureus*, while benzylpenicillin encountered resistance in 8 cases involving *Streptococcus pneumoniae*. Lower levels of resistance were noted for gentamicin and vancomycin, with seven and five resistant cases, respectively. *Pseudomonas aeruginosa* was the primary resistant pathogen for gentamicin, while *Staphylococcus aureus* displayed resistance to vancomycin, which is particularly alarming given that vancomycin is a critical option for treating resistant Gram-positive infections (Table 8).

Logistic regression analysis identified several significant predictors of chronic otomastoiditis outcomes. Delayed diagnosis (duration of symptoms > 6 months) was associated with a higher likelihood of complications, including cholesteatoma (OR: 3.8, 95% CI: 2.1–6.5, *p* < 0.001). Rural origin also emerged as a significant predictor of delayed presentation and increased disease severity (OR: 2.5, 95% CI: 1.4–4.3, *p* = 0.003). Furthermore, antibiotic resistance, particularly to amoxicillin, was independently associated with poorer treatment outcomes (OR: 3.1, 95% CI: 1.9–5.0, *p* < 0.001). In this study, socioeconomic factors were identified as significant contributors to delayed diagnosis and treatment of chronic otomastoiditis. Patients from rural areas accounted for 39.73% of cases and were more likely to present with advanced disease compared to urban patients (60.27%). Among rural patients, a higher percentage had a disease progression exceeding one year (65%) compared to urban patients (35%), indicating a correlation between rural residency and delayed healthcare access. Additionally, educational levels were linked to disease severity: patients with primary or secondary education comprised 70% of the cases with advanced complications, while only 10% of the patients with higher education reported similar progression. Employment status further influenced outcomes, with unemployed individuals and retirees representing 32.86% of the cases with delayed presentations, likely due to limited financial resources and restricted access to healthcare services.

These findings highlight the critical need for early diagnosis and targeted antibiotic stewardship, particularly in resource-limited settings (Table 9).

## 4. Discussion

Otomastoiditis is an inflammatory condition of the middle ear and mastoid air cells, typically arising from chronic otitis media. In this 10-year study of 292 patients, chronic cases were observed, frequent in urban areas (60.27%) compared to rural regions (39.73%). Factors such as delayed treatment, improper antibiotic use, and socioeconomic conditions, particularly in rural populations, contributed to the higher prevalence of chronic cases. These findings align with the 2011 Dolj County census, which showed a higher proportion of urban residents (52.1%) compared to rural (47.9%), a trend that has continued in subsequent years. The patients from rural areas tended to present later, often with more severe complications, due to factors like distance and delayed medical consultation. In contrast, 60.27% of the patients were from urban areas, where easier access to healthcare and better health education played a role. Otomastoiditis cases were less frequently observed in children under 10 years of age and adults over 70, with these populations often presenting notable complications. In the elderly patients over 70, decreased immunity and chronic conditions led to complicated otomastoiditis with poor treatment response. Males (56.16%) had a slightly higher incidence than females. The disease was more prevalent in young adults and middle-aged patients, particularly in those with lower education levels and retirees with chronic conditions. The rise in otomastoiditis among young adults (20–30 years) is linked to improper outpatient treatments and antibiotic resistance, leading to the spread of infection and prompting patients to seek medical care. Similar trends have been observed in other studies conducted over 10 or 20 years, which also report a rising incidence of chronic otomastoiditis in this age group [28].

The most common symptoms were hearing loss, fetid otorrhea, and otalgia. Chronic otomastoiditis, often involving cholesteatoma (49.31%), was marked by long-term ear discharge, high prevalence of chronic otomastoiditis reflects global patterns and is linked to delayed treatment and improper antibiotic use, as discussed by Nussinovitch et al. [18,29].

Suppurative otomastoiditis remains a relevant topic due to the functional and vital disturbances it causes. Several recent publications have highlighted an increase in the incidence of chronic mastoiditis, suggesting a possible correlation between the incorrect use of antibiotics and the rising number of mastoid infections. From the analysis of these data, we observed a significant number of patients with chronic conditions, where an exacerbation prompted them to seek specialized medical services. Increasingly, other studies show that the use of antibiotics as self-medication or in incomplete courses has led to the emergence of a new pathology by transforming acute otitis into latent otitis with an insidious progression, which promotes the occurrence of complications, primarily through the spread of infection to the mastoid cavities. Consequently, otomastoiditis frequently develops with highly virulent pathogens following prolonged antibiotic treatments [30,31].

The 10–20-year progression of the disease explains the findings from the hearing tests, tonal audiograms, and radiological examinations, which showed mixed hearing loss and the extensive destruction of the middle and inner ear structures. Most of the patients were in the chronic stage, and similar results have been reported in long-term studies, highlighting that pro-longed disease progression is a key factor in the onset of deafness. This condition significantly impacts the patient’s social interactions and overall quality of life. In his study, Maroldi R. details the radio-imaging aspects of otomastoiditis according to the stage of the disease, covering both the middle and inner ear, with specific mention of the features characteristic of the chronic stage, as outlined in this work’s chapter dedicated to imaging studies [32,33]. For instance, in his retrospective 12-year study, Anne S. describes and comments on the clinical symptomatology of chronic mastoiditis in children, noting the severe manifestations specific to this condition during childhood, such as high fever, abundant otorrhea, and the rapid onset of complications [34].

Chronic otomastoiditis features gradual hearing loss, minimal fetid otorrhea, and inconsistent pain. It is often associated with comorbidities like hypertension (39.04%) and diabetes (21.91%), reflecting findings by Morris et al., who linked these conditions to prolonged infection and resistance to standard therapies. The patients with comorbidities of ten presented with more severe disease, requiring tailored treatment approaches [35,36].

The location of tympanic perforation helps guide the diagnosis toward specific types of lesions. While epitympanic perforation is commonly associated with cholesteatoma, cholesteatoma can also occur in mesotympanic locations, depending on the retraction pocket’s position. Mesotympanic perforations are more frequently observed in suppurative and polypoid forms, whereas quasi-total perforation is typically seen in clinical forms with associated otic lesions, as corroborated by other studies in the field. In his research study, Mansour S. mentioned a similarly high number of chronic suppurative oto-mastoiditis cases. Moreover, cholesteatomatous forms were the most frequently diagnosed, a finding consistent with our study [37].

In our study, the most numerous anatomoclinical forms of otomastoiditis were cholesteatomatous (49.31%). Cholesteatomas can be considered benign tumors characterized by the abnormal growth of epithelial cells lining the middle ear and mastoid cavities, associated with complex and dynamic changes in the cells of the corium and extracellular matrix. Postoperative recovery was generally favorable in the patients without major complications, with the majority discharged within two weeks. However, in the cases involving intra-cranial complications or extensive cholesteatoma, a longer hospitalization period was required.

*Streptococcus pneumoniae* (36.56%) and *Staphylococcus aureus* (13.43%) were the pre-dominant pathogens; their significant resistance to amoxicillin and amoxicillin–clavulanate highlights the growing challenge of antibiotic resistance, as noted in Bonko et al. research [38]. Treatment approaches were determined based on the severity and chronicity of the condition. Antibiotic therapy was the first line of management; however, resistance to commonly used antibiotics, such as amoxicillin and amoxicillin–clavulanate, was noted, particularly against *Streptococcus pneumoniae* and *Pseudomonas aeruginosa*. Surgical interventions, including mastoidectomy, were performed in patients with advanced disease or complications like cholesteatoma, which accounted for a significant portion of the cohort (49.31%). Resistance to first-line antibiotics such as amoxicillin (32 cases) and amoxicillin–clavulanate (23 cases) underscores the urgent need for alternative treatment strategies. Similar patterns of resistance were reported by Hoberman et al., further emphasizing the global challenge of antibiotic resistance in chronic otomastoiditis [39]. Recent studies have similarly observed high levels of resistance in these pathogens, particularly in chronic and recurrent cases of otitis media. For instance, a study by Duff et al. emphasized the increasing difficulty in treating ear infections due to emerging multidrug-resistant strains of Streptococcus pneumoniae, which complicates empirical antibiotic therapies [40]. Moreover, research by Alshehri et al. found that chronic cases of otomastoiditis, especially those with cholesteatoma, require more aggressive treatment strategies due to the higher likelihood of complications and poorer response to standard antibiotics, which is consistent with our study’s findings that a significant proportion of chronic cases are cholesteatomatous [41]. In addition, findings from Nshimirimana et al. demonstrated similar trends regarding the delayed presentation of rural patients, leading to more severe complications at the time of diagnosis, underscoring the importance of healthcare accessibility in managing chronic ear infections [42]. Finally, recent developments in diagnostic and therapeutic approaches suggest that high-resolution imaging and earlier surgical interventions, such as mastoidectomy, are increasingly being recommended in cases with significant antibiotic resistance and complicated cholesteatomas, corroborating our study’s emphasis on surgical management for such cases [43]. Delayed diagnosis and rural origin were identified as significant contributors to disease severity and complications, underscoring the importance of improving healthcare access and early intervention in underserved populations. Additionally, the association between antibiotic resistance and poor outcomes reinforces the need for optimized antibiotic stewardship programs and the development of novel therapeutic strategies to combat multidrug-resistant infections. The recurrence rates observed in this study emphasize the need for individualized postoperative management strategies. Patients with higher risk factors, such as comorbidities or antibiotic-resistant infections, require closer follow-up and tailored interventions to mitigate recurrence risks. The timing of recurrences, predominantly occurring within the first two years post-surgery, suggests that intensive monitoring during this period is critical. Long-term management strategies should include routine imaging, audiological assessments, and personalized rehabilitation protocols. Improved patient outcomes require a multidisciplinary approach that combines surgical expertise, infectious disease management, and comprehensive postoperative care. This is particularly crucial in resource-limited settings, where delayed diagnosis and limited access to advanced treatment options exacerbate disease severity. Enhanced follow-up protocols, incorporating imaging and audiological evaluations, can help detect and manage recurrence early, reducing long-term complications. Furthermore, collaborative public health efforts focused on education, prevention, and accessible healthcare services are essential to address the broader determinants of antibiotic resistance and its impact on otomastoiditis management.

One of the major strengths of this study is its comprehensive analysis of chronic otomastoiditis cases over an extended 10-year period. This allows for a robust assessment of the clinical, demographic, and microbiological characteristics of a large and diverse patient cohort, providing valuable insights into the continuum of disease progression. Additionally, the inclusion of bacteriological studies and antibiotic resistance patterns offers a practical evaluation of current treatment challenges, especially in the context of rising antibiotic resistance. The study’s focus on both surgical and medical outcomes and further strengthens its clinical relevance, particularly in cases complicated by cholesteatoma, which is an important area of concern for both otolaryngologists and infectious disease specialists. However, there are several limitations to consider. First, this is a retrospective study, which inherently limits the ability to control for confounding variables and introduces potential selection bias due to incomplete patient records. Another limitation is that this study was conducted in a single tertiary care center, which may limit the generalizability of the findings to other populations, particularly in primary or rural healthcare settings. Finally, the length of hospitalization was influenced by institutional protocols during the time of the study, which may not reflect the current best practices in all clinical environments. Antibiotic resistance poses a significant challenge in the management of chronic otomastoiditis, particularly with the rising resistance to first-line antibiotics such as amoxicillin and amoxicillin–clavulanate. This resistance has been shown to directly impact treatment outcomes, often necessitating the use of second-line therapies, including fluoroquinolones or third-generation cephalosporins. Such changes in therapeutic approaches increase the complexity and cost of care while contributing to the global burden of multidrug-resistant infections. These clinical implications extend to delayed recovery, higher recurrence rates, and an increased need for surgical interventions in affected patients. Improved patient outcomes require a multidisciplinary approach that combines surgical expertise, infectious disease management, and comprehensive postoperative care. This is particularly crucial in resource-limited settings, where delayed diagnosis and limited access to advanced treatment options exacerbate disease severity. Enhanced follow-up protocols, incorporating imaging and audiological evaluations, can help detect and manage recurrence early, reducing long-term complications. Furthermore, collaborative public health efforts focused on education, prevention, and accessible healthcare services are essential to address the broader determinants of antibiotic resistance and its impact on otomastoiditis management.

Although a prospective or randomized controlled trial (RCT) might provide stronger evidence regarding causality and intervention outcomes, such designs were not feasible for this study due to ethical and logistical considerations. The retrospective nature of this research allowed for the identification of patterns in disease progression, healthcare accessibility, and antibiotic resistance in a real-world setting, which is particularly relevant given the public health implications of chronic otomastoiditis. Future research should consider prospective or controlled designs to complement the findings presented here and further elucidate causal relationships. Other directions in managing chronic otomastoiditis include the integration of emerging diagnostic and therapeutic technologies. High-resolution imaging techniques, such as computed tomography (CT) and magnetic resonance imaging (MRI), can aid in the early detection of complications, improving the precision of both medical and surgical interventions. Additionally, novel antibiotic formulations and adjunctive therapies, such as antimicrobial peptides, hold promise in overcoming resistance and enhancing treatment efficacy. Research into phage therapy and other alternative modalities also offers potential for addressing the infections caused by multidrug-resistant organisms.

## 5. Conclusions

Chronic otomastoiditis remains a prevalent issue, with delayed medical intervention, particularly in rural populations, contributing to the severity of disease presentations and complications. This study highlights the substantial impact of antibiotic resistance on otomastoiditis management, with *Streptococcus pneumoniae* and *Staphylococcus aureus* identified as the most common pathogens. Notably, these pathogens demonstrated significant resistance to commonly prescribed antibiotics such as amoxicillin and amoxicillin–clavulanate, especially among *Pseudomonas aeruginosa* and *Staphylococcus aureus* isolates. These findings underscore the necessity for early and accurate diagnosis alongside targeted antibiotic therapy to mitigate the progression of chronic cases. Surgical treatment, particularly mastoidectomy, plays a critical role in managing advanced cases and associated complications. A combined approach involving timely diagnosis, effective antibiotic stewardship, and appropriate surgical intervention is crucial to prevent long-term complications, including hearing loss and extensive bone damage. This study also emphasizes the need for public health strategies focused on improving healthcare accessibility and patient education, especially in underserved rural areas. Enhancing access to specialized care and promoting early intervention could significantly alleviate the chronic burden of otomastoiditis and reduce the incidence of severe complications. Our findings highlight socioeconomic factors, delayed diagnoses, and antibiotic resistance as primary predictors of complications, indicating an urgent need for revised empirical treatment protocols, particularly in resource-limited settings.

## Figures and Tables

**Table 1:** Distribution of patients by age group, gender and place of origin.

Age	Men	Women	Rural	Urban
0–10 years	6	4	4	6
11–20 years	22	12	14	20
21–30 years	32	26	24	34
31–40 years	30	18	20	28
41–50 years	20	22	16	26
51–60 years	34	30	22	42
61–70 years	16	14	12	18
71–80 years	4	2	4	2

**Table 2:** Numerical distribution of patients by gender, place of origin, and disease duration.

	<3 Months		>3 Months		Total
Men	10	3.42%	152	52.05%	162
Women	6	2.05%	124	42.46%	130
Rural	12	4.05%	104	35.61%	116
Urban	4	1.36%	172	58.90%	176

**Table 3:** Distribution of patients with otomastoiditis by anatomoclinical forms.

Anatomoclinical Forms	Number of Patients	Percentage
Simple Cholesteatomatous	10	3.42%
Suppurative Cholesteatomatous	110	37.67%
Simple Polypoid	16	5.47%
Suppurative Polypoid	52	17.80%
Simple Suppurative	66	22.60%
Suppurative Polypoid Cholesteatomatous	38	13.01%

**Table 4:** Distribution of patients by anatomoclinical form and duration of hospitalization.

Duration of Hospitalization	Cholesteatomatous Form		Polypoid Form		Suppurative Form		Cholesteatomatous-Polypoid-Suppurative Form		Total	
<2 weeks	98	81.67%	62	91.18%	62	96.88%	16	40.00%	238	81.51%
>2 weeks	22	18.33%	6	8.82%	2	3.13%	24	60.00%	54	18.49%

**Table 5:** Imaging characteristics and complications by otomastoiditis type.

Otomastoiditis Type	Imaging Findings	Complications Observed	Prevalent Age Group
Cholesteatomatous	Bone erosion, cholesteatoma	Labyrinth involvement	30–50
Polypoid	Soft tissue swelling	No complications	20–40
Suppurative	Mastoid air cell opacification	Intracranial spread	40–60

**Table 6:** Surgical outcomes and postoperative recurrence rates.

Surgical Intervention	Total Patients (%)	Complications (%)	Recurrence Rate (Within 24 Months)	Recovery Rate (%)
Mastoidectomy	68% (198)	21.7% (43)	18.5%	76.3%
Tympanoplasty + Mastoidectomy	32% (94)	14.9% (14)	10.6%	83.2%
Simple tympanoplasty	8% (24)	8.3% (2)	4.2%	91.7%
Revision surgery (recurrent cases)	5% (15)	33.3% (5)	26.7%	73.3%
Total	100% (292)	20.2% (59)	15.2%	79.1%

**Table 7:** Distribution of identified pathogens by number of cases and percentage.

Pathogen	Number of Cases	Percentage
*Pseudomonas aeruginosa*	30	11.19%
*Streptococcus pneumoniae*	98	36.56%
*Staphylococcus aureus*	36	13.43%
*Proteus mirabilis*	14	5.22%
*Haemophylus influenzae*	18	6.71%
No growth detected	32	11.94%

**Table 8:** Antibiotic resistance in pathogens isolated from patients with chronic otomastoiditis.

Antibiotic	Number of Patients with Resistance	Resistant Pathogens
amoxicillin	32	*Streptococcus pneumoniae*, *Staphylococcus aureus*
amoxicillin–clavulanate	23	*Pseudomonas aeruginosa*, *Staphylococcus aureus*
ceftriaxone	16	*Haemophilus influenzae*, *Proteus mirabilis*
ciprofloxacin	13	*Pseudomonas aeruginosa*, *Staphylococcus aureus*
azithromycin	11	*Staphylococcus aureus*
benzylpenicillin	8	*Streptococcus pneumoniae*
gentamicin	7	*Pseudomonas aeruginosa*
vancomycin	5	*Staphylococcus aureus*

**Table 9:** Risk factors and odds ratios for developing complications in chronic otomastoiditis.

Risk Factor	Odds Ratio	95% Confidence Interval	*p*-Value
Delayed diagnosis (>6 months)	3.8	2.1–6.5	<0.001
Rural origin	2.5	1.4–4.3	0.003
Recurrent antibiotic use	3.1	1.9–5.0	<0.001
Presence of diabetes	2.2	1.2–4.1	0.01

## Data Availability

Data are contained within the article.
